# Production of Functional Buttermilk and Soymilk Using *Pediococcus acidilactici* BD16 (*alaD*^+^)

**DOI:** 10.3390/molecules26154671

**Published:** 2021-08-02

**Authors:** Anshula Sharma, Masafumi Noda, Masanori Sugiyama, Ajaz Ahmad, Baljinder Kaur

**Affiliations:** 1 Systems Biology Laboratory, Department of Biotechnology, Punjabi University, Patiala 147002, India; anshula_rs17@pbi.ac.in; 2 Graduate School of Biomedical and Health Sciences, Hiroshima University, Kasumi, Minami-ku, Hiroshima 734-8553, Japan; bel@hiroshima-u.ac.jp (M.N.); sugi@hiroshima-u.ac.jp (M.S.); 3 Department of Clinical Pharmacy, College of Pharmacy, King Saud University, Riyadh 11451, Saudi Arabia; aajaz@ksu.edu.sa

**Keywords:** bioactive metabolites, functional drinks, L-alanine, LAB fermentation, metabolomic fingerprinting, *Pediococcus acidilactici*, starter culture bacteria

## Abstract

Functional foods or drinks prepared using lactic acid bacteria (LAB) have recently gained considerable attention because they can offer additional nutritional and health benefits. The present study aimed to develop functional drinks by the fermentation of buttermilk and soymilk preparations using the *Pediococcus acidilactici* BD16 (*alaD*^+^) strain expressing the L-alanine dehydrogenase enzyme. LAB fermentation was carried out for 24 h and its impact on the physicochemical and quality attributes of the fermented drinks was evaluated. Levels of total antioxidants, phenolics, flavonoids, and especially L-alanine enhanced significantly after LAB fermentation. Further, GC-MS-based metabolomic fingerprinting was performed to identify the presence of bioactive metabolites such as 1,2-benzenedicarboxylic acid, 1-dodecene, 2-aminononadecane, 3-octadecene, 4-octen-3-one, acetic acid, azanonane, benzaldehyde, benzoic acid, chloroacetic acid, colchicine, heptadecanenitrile, hexadecanal, quercetin, and triacontane, which could be accountable for the improvement of organoleptic attributes and health benefits of the drinks. Meanwhile, the levels of certain undesirable metabolites such as 1-pentadecene, 2-bromopropionic acid, 8-heptadecene, formic acid, and propionic acid, which impart bitterness, rancidity, and unpleasant odor to the fermented drinks, were reduced considerably after LAB fermentation. This study is probably the first of its kind that highlights the application of *P. acidilactici* BD16 (*alaD*^+^) as a starter culture candidate for the production of functional buttermilk and soymilk.

## 1. Introduction

In recent years, mounting public awareness regarding diet-associated health issues has led to the development of natural and healthy functional foods or dietary items. Functional foods or drinks provide additional nutrients and energy to beneficially modulate metabolic functions of the body and reduce the risks associated with certain diseases [1].

Functional soymilk is a milky fluid obtained from the overnight soaked and mashed soybeans, which contains many bioactive components that can reduce the pathophysiological responses associated with gut disorders to improve human health [2]. Soy-based foods or drinks are inexpensive and rich nutritional supplements for vegetarian and lactose-intolerant people. Previous studies have also reported that these are abundant in isoflavones and high-quality proteins that help in reducing the risks of Alzheimer’s disease, atherosclerosis, cancer, cardiovascular and hepatic disorders, diabetes, irregular menstrual cycles and menopausal syndromes, and premature aging. Moreover, they also assist in immunomodulation, neuroprotection, weight management, and enhanced athletic performance [3,4,5]. The aglycone form of isoflavones is preferred over the glycosidic form due to its enhanced bioavailability and health-imparting attributes. Thus, fermentation of soymilk with a suitable LAB strain is suggested to make isoflavones more bioavailable in the human body and to retrieve its maximum health benefits. Intestinal LAB strains have the capability of producing the β-glucosidase enzyme that hydrolyzes β-glycosidic linkages present in the isoflavones and results in the production of aglycone compounds, eventually enhancing their antioxidant and anti-tumor properties [4]. Moreover, LAB -mediated fermentation of soymilk also eliminates several non-digestible oligosaccharides by secreting the α-galactosidase enzyme [6]. LAB fermentation also mediates the production of several bioactive peptides such as antibacterial, antithrombotic, hypotensive, immunomodulatory, mineral binding, and opioid peptides that play a significant role in the prevention of various health disorders [7]. In addition, fermenting soymilk with a suitable LAB strain also improves its sensory attributes by removing undesirable beany flavor [5].

Buttermilk is another very popular and desirable nutritional drink for lactose intolerant individuals, produced as a byproduct from the churning of the unfermented milk cream [8]. Buttermilk is also a rich source of proteins, vitamins, milk salts, and nutritional ingredients [9]. Currently, a wide range of buttermilk variants are available in the market but cannot be considered as health drinks due to added sugar, flavors, and preservatives in them, which can further impose serious health problems. Therefore, the preparation of functional buttermilk and soymilk using a natural flavor and a nutrition-imparting LAB strain is highly recommended to reduce their undesirable flavor and enhance consumer acceptability.

L-alanine is an FDA-approved food flavor, low-calorie sweetener, fat substitute, and nutritional supplement [10,11]. It is an ideal ingredient for developing pharmaceutics to treat hypoglycemia, prostate hypertrophy, liver diseases, and urea cycle disorders [12,13,14]. It is widely used in fragrances, hair and skin conditioners, and personal care products [15,16]. Moreover, it provides high energy during intensive workouts, thus is added as a supplement to energy drinks to enhance athletic performance [17]. Therefore, an L-alanine producing *P. acidilactici* BD16 (*alaD*^+^) strain is being proposed hereby as a starter culture bacterium for the production of functional buttermilk and soymilk. Fermentation of buttermilk and soymilk was carried out for 24 h and evaluated for variations in their physiochemical and quality attributes. Further, GC-MS-based metabolomic fingerprinting was performed to identify the effect of LAB fermentation on increasing levels of certain desirable metabolites with pharmacological and flavor enhancement properties and reducing undesirable and quality detrimental compounds that impart bitterness, unpleasant flavor, rancidity, and unhealthy attributes to the fermented drinks.

## 2. Materials and Methods

### 2.1. Pediococcus acidilactici BD16 (alaD^+^) Strain and Culture Conditions

A synthetic *alaD* gene encoding alanine dehydrogenase enzyme, which catalyzes the reductive amination of pyruvate to L-alanine, was designed insilico and further cloned into the pLES003 vector and expressed using an auto-inducible P_289_ promoter (unpublished data). The pLES003 vector is a shuttle vector that can replicate in both Gram-negative *E. coli* as well as in Gram-positive lactic acid bacteria [18,19]. The sequence of the synthetic alaD gene construct was submitted to the Genbank database under the accession number MT108231. The recombinant pLES003alaD vector was further introduced into a native *P. acidilactici* BD16 (MTCC 10973) strain using standard DNA transformation procedures as mentioned earlier by Kaur and co-workers [18]. The *P. acidilactici* BD16 (*alaD*^+^) harboring pLES003alaD vector was further revived in de Man’s Rogosa Sharpe Medium (MRS) broth containing dextrose 20 g/L, beef extract 10 g/L, peptone 10 g/L, sodium acetate 5 g/L, yeast extract 5 g/L, tri-ammonium citrate 2 g/L, dipotassium hydrogen phosphate 2 g/L, magnesium sulfate 0.1 g/L, manganous sulfate 0.05 g/L, erythromycin 20 μg/mL, and Tween 80 1 mL/L, pH 6.5 ± 0.2 undermicroaerophilic and stationary conditions at 37 °C for 24 h [20]. After revival, 2% *v*/*v* culture broth grown overnight (containing 10^6^ cfu/mL) was used as an inoculum for the fermentation experiments. Antibiotic pressure was maintained during the revival and subculturing of the recombinant *P. acidilactici* (*alaD*^+^) bearing pLES003alaD vector to maintain its copy number. Keeping in view the food safety aspects, antibiotic addition was avoided during further experiments such as inoculum preparation and fermentation of the drinks.

### 2.2. Procurement of Buttermilk and Preparation of Soymilk Samples

Buttermilk without added flavor and preservatives was procured from Verka Milk Plant, Patiala, Punjab, India. For the preparation of fresh soymilk, an adequate number of whole soybeans was taken, washed, and soaked in distilled water overnight at room temperature. The next day, the water was decanted, and the soaked soybeans were mixed with distilled water (approximately 3 times their weight) and mashed using a mechanical blender. Afterward, the slurry was filtered using a muslin cloth and a milky-looking filtrate (soymilk) was collected. Raw soymilk was sterilized by heating at 115 °C for about 10 min [21]. Soymilk was then cooled at an ambient temperature and stored in the refrigerator at 4 °C for further experimentation [5,22].

### 2.3. Fermentation of Buttermilk and Soymilk Using P. acidilactici BD16 (alaD^+^)

For the fermentation of buttermilk and soymilk, 80 mL of each sample was taken into 100 mL sterile Erlenmeyer flasks and inoculated using 2% *v*/*v* inoculum (containing 10^6^ cfu/mL), followed by incubation at 37 °C for 24 h under stationary conditions. Un-fermented (un-inoculated but incubated under similar conditions) drinks were used as controls for comparative analysis. Higher volumes of buttermilk and soymilk in the culture flasks have been used intentionally to maintain the oxygen-deprived conditions.

### 2.4. Analysis of Bacterial Growth during Fermentation of Buttermilk and Soymilk

Buttermilk and soymilk samples were inoculated using a requisite amount of *P. acidilactici* BD16 (*alaD*^+^) culture and kept at 37 °C for 24 h under microaerophilic and stationary conditions as described above. During fermentation, the growth profile of the LAB strain was studied by determining cell density in terms of cfu/mL. For this, milk solids were removed by filtration and the clear filtrate was then appropriately diluted before plating onto MRS agar plates for enumeration of the bacterial counts.

### 2.5. Variation in pH and Milk Consistency during LAB Fermentation

Variation in pH was recorded every 2 h during LAB fermentation of the samples. The pH of test samples was determined using a digital pHmeter after its calibration using buffer solutions of pH 4 and 7 at ambient temperature. Variations in the milk consistency were also recorded after 2 h interval to evaluate the optimum fermentation period.

### 2.6. Estimation of In-Situ L-Alanine Production

In-situ L-alanine production during fermentation of buttermilk and soymilk was estimated after regular intervals (after every 2 h) for 24 h [23]. For this, 1 mL of each sample was taken, centrifuged at 12,000× *g* for 5 min to obtain the supernatant. To the supernatant, 5 mL of Dichlone reagent (62.5 mg) prepared in dimethyl sulphoxide was added and kept for incubation in a boiling water bath for 10 min to allowthe development of orange color. The reaction mixture was allowed to cool down and was further diluted in 94 mL hydrochloric acid (0.5 M). Thereafter, the absorbance of each sample was noted at 470 nm against a blank (containing sodium bicarbonate and Dichlone reagent only) and L-alanine concentrations were determined using the standard curve of L-alanine [23]. The concentration of L-alanine in unfermented buttermilk and soymilk samples was also estimated for comparison. The enantiomeric purity of the in situ produced L-alanine was estimated by analyzing its specific optical rotation using a polarimeter [24].

### 2.7. Determination of Physicochemical and Quality Attributes of Buttermilk and Soymilk during the Course of Fermentation

#### 2.7.1. Estimation of Total Solids and Moisture Content

The total solids and moisture content of buttermilk and soymilk were determined twice (before and after fermentation) according to the procedure described by Jiang, Cai and Xu [2]. For this, approximately 10 mL of each sample was placed into a pre-weighed crucible, weighed, and further placed into a hot air oven set at 100 °C for 15 h. Later, dried residues were weighed after cooling using a desiccator to calculate the total solids and moisture content, presented in the terms of percentage.

#### 2.7.2. Estimation of Total Protein Concentration and Titratable Acidity

Protein concentrations in the unfermented and fermented buttermilk and soymilk were determined using the Lowry method [25]. Titratable acidity (TA) was calculated in terms of %age lactic acid, by titration of the test samples against 0.1 N NaOH solution using phenolphthalein as an indicator to allow the development of light pink color [26]. TA was further calculated using the following Equation (1):

(1) $$\mathrm{TA}\;\left(\%\right)=\frac{a\times f\times 0.09}{m}$$

where a = consumed volume (mL) of 0.1 N NaOH;

f = 0.1 N NaOH factor;
m = volume of test sample (mL)

#### 2.7.3. Estimation of Carbohydrate Content

Carbohydrate contents in both fermented and unfermented buttermilk and soymilk were estimated using the standard anthrone method. For this, 1 mL of each test sample was mixed with 5 mL of the anthrone reagent, followed by incubation in a boiling water bath for 10 min. A furfural compound was formed due to dehydration of the carbohydrates by concentrated sulfuric acid and, further, its reaction with the anthrone reagent resulted in the formation of a blue–green colored complex. The absorbance was taken at 620 nm and the carbohydrate concentration was calculated from the standard curve, and presented in the terms of percentage [27,28].

#### 2.7.4. Estimation of Antioxidant Activity

The antioxidant activity of the samples was analyzed using the 1,1-diphenyl-2-picrylhydrazyl (DPPH) scavenging activity assay as described by Fakri and coworkers [29]. For the antioxidant assay, 900 μL ethanolic solution (96%) with or without DPPH (0.1 mM) was mixed with 100 μL of the test sample. The mixture was vortexed and incubated at room temperature in the dark for 30 min. After incubation, the absorbance of the solution was measured at 520 nm using a UV-VIS spectrophotometer against water as a blank. Ascorbic acid was used as standard, and the results were expressed as a percentage (%) of DPPH scavenging activity calculated as follows:

(2) $$\%\;\mathrm{inhibition}=\frac{\left(\mathrm{Absorbance}\;\mathrm{of}\;\mathrm{control}-\mathrm{Absorbance}\;\mathrm{of}\;\mathrm{test}\;\mathrm{sample}\right)\times 100}{\mathrm{Absorbance}\;\mathrm{of}\;\mathrm{control}}$$

#### 2.7.5. Estimation of Total Phenolic Content

The total phenolic content (TPC) of the unfermented and fermented buttermilk and soymilk was determined using the standard method described by Fakri and coworkers [29]. For the TPC assay, 0.1 mL of test samples were first mixed with 0.9 mL of distilled water. Further, 1 mL of the Folin–Ciocalteu reagent (diluted in 1:2 ratio in distilled water) and 2 mL of 10% sodium carbonate solution (Na_2_CO_3_) were added to the test sample mixtures, followed by incubation at room temperature for about 2 h to allow the development of the blue color. Samples were centrifuged, supernatants were collected in fresh vials, and their absorbance was measured at 765 nm. TPC was determined using a standard curve of gallic acid and the results were expressed in terms of gallic acid equivalent (mg GAE/mL sample).

#### 2.7.6. Estimation of Total Flavonoid Content

The total flavonoid content (TFC) of the samples was determined using a colorimetric method as described by Ma and Huang [30]. For the TFC assay, 1 mL of each test sample was mixed with 150 μL of 5% sodium nitrate solution. Further, 300 μL of the 10% aluminum chloride solution and 1 mL of 1 M NaOH were added to the reaction mixture. Finally, distilled water was added to fix the solution to 3.5 mL. The absorbance of the samples was measured immediately at 510 nm against the blank. TFC was calculated using a quercetin standard and the results were expressed as milligrams of quercetin equivalent (mg quercetin/mL sample).

### 2.8. Sensory Evaluation

Sensory evaluation was carried out to analyze the enhancement of various sensory attributes and overall acceptance of the fermented drinks. Sensory analysis of the fermented drinks was performed using a panel of 10 healthy volunteers, and variations were recorded in terms of hedonic scale parameters and radar graphs [31,32]. Homogenized samples of the unfermented and fermented drinks were tested for characteristics such astaste, odor, mouthfeel, and undesirable flavor. The 9-point hedonic scale was used to evaluate where the degree of acceptance (between extremely liked and extremely disliked). Based on the observed data, radar graphs were drawn to compare the parametric differences between unfermented and fermented drinks.

### 2.9. GC-MS Based Metabolomic Fingerprinting for Identification of Bioactive and Health-Promoting Compounds

#### 2.9.1. Sample Preparation for GC-MS

GC/MS analysis of the unfermented and fermented buttermilk and soymilk was performed for comparative metabolomic fingerprinting. For this, 1 mL of each was drawn and was extracted using ethyl acetate and centrifuged at 5000 rpm for 5 min. After centrifugation, the upper layer was separated carefully and transferred to a fresh Eppendorf for its derivatization. The derivatization reagent consisting a mixture of *N*,*O*-bis (trimethylsilyl) trifluoroacetamide (BSTFA):ethyl acetate: pyridine (prepared in the ratio of 3:1:1 *v*/*v*/*v*) was added to the samples in an equal amount. Incubation was performed at 37 °C for 24 h. Ethyl acetate extraction and further derivatization using BSTFA facilitate the chromatographic separation of analytes by reducing polarities of the functional groups [33].

#### 2.9.2. GC-MS Analysis

The prepared samples were then injected manually in the Gas Chromatography system equipped with a mass spectrometer (Perkin Elmer Clarus 500, Waltham, MA, USA). GC/MS analysis was carried out using Rxi-5 Sil MS column (0.25 μm × 30 m × 0.25 mm) operating under the electron impact mode at 70 eV, in the presence of helium as a carrier gas, used at a flow rate of 1 mL/min, and with an injection volume of 1 μL. The oven injector temperature was gradually raised from 50 °C to 200 °C with an increase of 8 °C/min, and then to 290 °C with an increase of 7 °C/min. Mass spectra of the fragments (with masses ranging from 40 to 550 Da) were recorded at 70 eV with a scan interval of 0.5 s. The mass spectra of the separated compounds were compared with the reference datasets stored in the NIST database for further identification of various compounds [2]. The variations in the biochemical composition of the unfermented and fermented drinks were recorded in terms of fold changes calculated using standard formulaas proposed by Mariani and co-workers [34].

(3) $$\mathrm{Fold}\;\mathrm{change}=\frac{\mathrm{PH}\;\mathrm{after}\;\mathrm{secondary}\;\mathrm{fermentation}-\mathrm{PH}\;\mathrm{before}\;\mathrm{secondary}\;\mathrm{fermentation}}{\mathrm{PH}\;\mathrm{before}\;\mathrm{secondary}\;\mathrm{fermentation}}$$

where PH = Peak height.

### 2.10. Statistical Analysis

All the estimations were performed in triplicate (three samples were taken and were analyzed individually) and the estimated records were averaged to calculate the values of mean, standard deviation, and standard error. *p*-value was calculated using theChi-square test to substantiate the statistical significance of the data obtained.

## 3. Results

### 3.1. Production of Functional Buttermilk and Soymilk Using P. acidilactici BD16 (alaD^+^)

The present study aims at the development of functional drinks using *P. acidilactici* BD16 (*alaD*^+^) with improved flavor, nutritional, and health-promoting characteristics. During the course of fermentation, changes in the bacterial growth profile, pH, acidity (in terms of lactic acid), and L-alanine production were recorded in the fermented drinks. Significant variations (*p* < 0.005) in the titratable acidity, total carbohydrates, proteins, flavonoids, phenolics, and antioxidant activity were observed and compared with their controls. The sensory attributes of the LAB fermented drinks such as flavor, odor, mouthfeel, and after-taste were also compared with the unfermented drinks. Results obtained in the study authenticate the claim of LAB fermentation in improving the nutritional and functional attributes of the drinks.

### 3.2. Analysis of Bacterial Growth during LAB Fermentation

**Figure 1:**
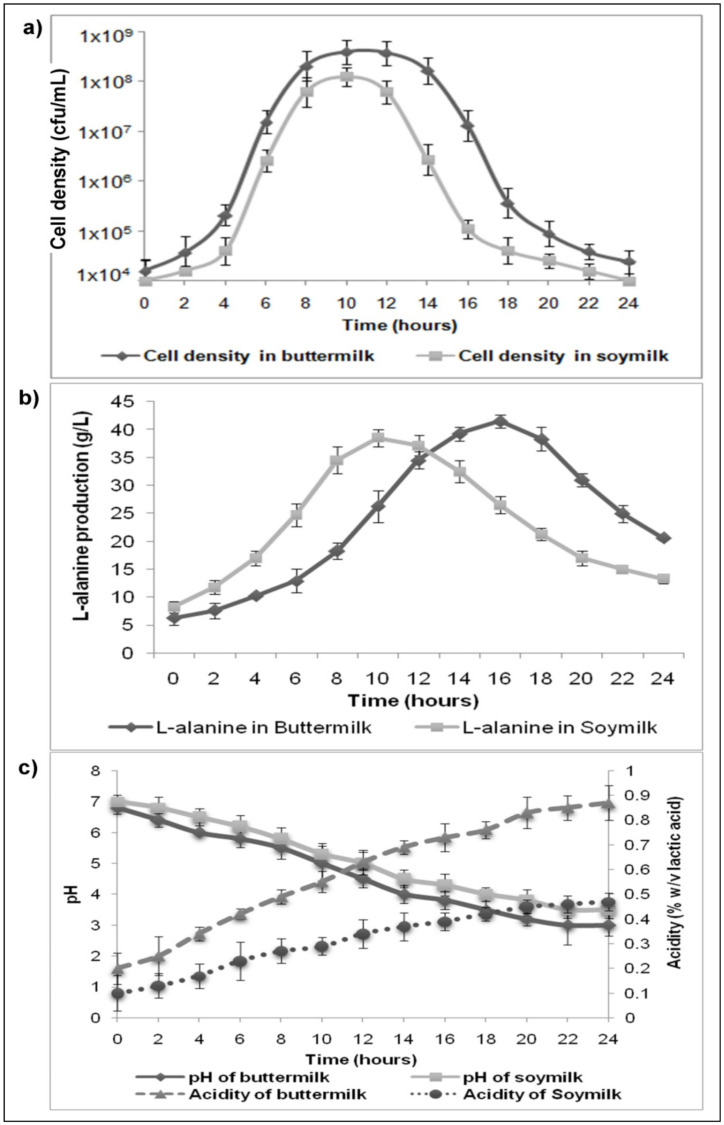
(**a**) Curves showing variations in cell densities (cfu/mL), (**b**) L-alanine production, and (**c**) pH vs. acidity in the buttermilk and soymilk during fermentation using *P. acidilctici* BD16 (*alaD*^+^). Values are presented as mean ± standard deviation (SD) of triplicate observations (n = 3).

The growth profile of *P. acidilactici* BD16 (*alaD*^+^) in buttermilk and soymilk samples was analyzed at regular intervals up to 24 h of fermentation, and a growth curve was plotted between the incubation time and the observed cell density in terms of cfu/mL (Figure 1a). The LAB strain achieved its maximum growth (in terms of highest cfu/mL) after 10 h of incubation. Almost similar growth patterns were observed in both drinks during the entire fermentation process.

A typical bacterial growth curve including lag, log, stationary, and decline phases was observed during fermentation. The strain experienced a luxurious growth as the cell biomass was enhanced drastically (from 10^4^ to 10^8^ cells) in comparison to the added inoculum, and the maximum growth was observed at 10 h of fermentation in both fermented drinks. After achieving maximum growth, the cell counts started declining subsequently due to the scarcity of the nutrients and enhanced production of secondary metabolites such as L-alanine and lactic acid.

### 3.3. Variations in pH and Milk Consistency during the Course of Fermentation

Fermented buttermilk and soymilk samples using the *P. acidilactici* BD16 (*alaD*^+^) strain showed a sharp drop in pH from 7 to 3 due to the accumulation of lactic acid (Figure 1c). The results of the present study showed that there was a fall in the pH and an increase in the acidity of the fermented drinks. This relationship between reduction in pH and increase in acidity is in accordance with the previous literature, where a drop in pH during LAB fermentation was associated with the accumulation of lactic acid [21,35]. In the case of soymilk, the formation of a soft gel was initiated due to curdling of the milk proteins after 6 h and the formation of a firm gel was noticed after 8–10 h of fermentation during which a major decline in pH was also observed. So, it indicates that the fermentation should be arrested between 6–7 h to stop further curdling of the soymilk that already has accumulated a significant amount of L-alanine. After fermentation, homogenization of the soymilk is recommended to obtain a smooth/creamy texture before storage at 4 °C. Buttermilk showed more pH variation and the final pH dropped from 6.8 to 3.0 during fermentation, whereas in the case of soymilk samples, the pH drop was recorded to be from 7 to 3.5.

### 3.4. In-Situ L-Alanine Production during Fermentation of Buttermilk and Soymilk

After LAB fermentation, the L-alanine content (with >97% enantiomeric purity) enhanced considerably in the buttermilk and soymilk preparations (as depicted in Figure 1b). L-alanine levels were enhanced 7-fold (from 6.75 ± 0.26 to 41.5 ± 0.5 g/L; 75 to 465 mM) in the buttermilk samples within 16 h of fermentation in comparison to the unfermented drink. Whereas, in the case of soymilk, L-alanine titers were increased 5-fold (from 8.25 ± 0.18 to 38.5 ± 0.39 g/L; 92 to 432 mM) within 10 h of fermentation in comparison to the unfermented drink. L-alanine titers started declining after 10 h of fermentation in the case of soymilk and after 16 h of fermentation in the case of buttermilk preparations. Thus, the authors strongly suggest restricting the period of soymilk fermentation up to 8 h and buttermilk fermentation up to 16 h, to further avoid settling of the milk solids or loss of nutrients. The recommended incubation periods are sufficient to impart a considerable level of sweetness (due to L-alanine) in the fermented drinks.

### 3.5. Physico-Chemical and Quality Attributes of Buttermilk and Soymilk during Fermentation of Buttermilk and Soymilk

**Figure 2:**
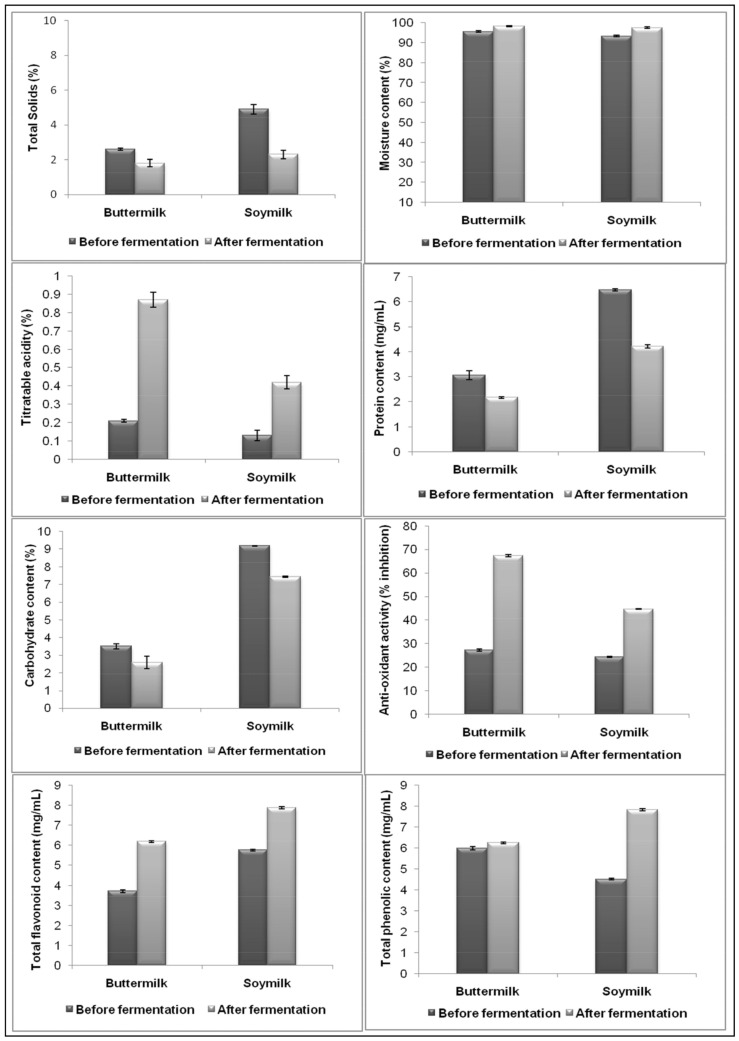
*P. acidilactici* BD16 (*alaD^+^*) induced variations inphysicochemical attributes of buttermilk and soymilk. Values are presented as mean ± standard deviation (SD) of triplicates (n = 3).

Fermentation using *P. acidilactici* BD16 (*alaD*^+^) greatly influenced many physicochemical and quality attributes of the buttermilk and soymilk preparations. Fermented buttermilk and soymilk showed enhanced contents of antioxidants, flavonoids, and phenols in comparison to the unfermented drinks, whereas the total solids, carbohydrates, and protein contents were reduced considerably upon LAB fermentation as discussed below (Table 1 and Figure 2).

#### 3.5.1. Variation in Physicochemical Parameters during the Course of Fermentation

Titratable acidity (TA) of the buttermilk and soymilk samples increased upon LAB fermentation due to the production of lactic acid. In the case of buttermilk, TA increased up to 0.87 ± 0.04% in comparison to the unfermented drink, which showed only 0.21 ± 0.02% TA, whereas the fermented soymilk showed a total acidity of 0.42 ± 0.03% as compared to the unfermented one with a TA of 0.13 ± 0.02% only. The total solid content of buttermilk and soymilk was also reduced substantially upon LAB fermentation. Buttermilk solid content decreased up to 1.8 ± 0.20% in comparison to the unfermented control, which had a total solid content of 2.6 ± 0.1%. In the case of soymilk, total solid content reduced considerably from 4.9 ± 0.35 to 2.3 ± 0.25%. Total solids were reduced after LAB fermentation possibly due to consumption, denaturation, and precipitation of the proteins triggered by lactic acid accumulation. Meanwhile, the moisture contents in the drinks increased after LAB fermentation. The moisture contents in the case of fermented buttermilk and soymilk increased from 95.6 ± 0.5 to 98.3 ± 0.31% and 93.4 ± 0.26 to 97.6 ± 0.40%, respectively. Previously, Sebastian, Barus, and Mulyono [21] also reported a reduction in the contents of total solids, crude protein, and an enhancement in the total moisture content upon fermentation of soymilk using *Lactobacillus delbrueckii* subsp. *bulgaricus* and *Streptococcus salivarius* subsp. *Thermophilus* strains.

#### 3.5.2. Variation in Protein and Carbohydrate Contents

The protein content of buttermilk was reduced from 3.06 ± 0.19 mg/mL (unfermented) to 2.17 ± 0.05 mg/mL (fermented). In the case of soymilk, protein content was 6.48 ± 0.06 mg/mL before fermentation, which decreased up to 4.21 ± 0.07 mg/mL after fermentation. The carbohydrate content of the buttermilk also was reduced from 3.5 ± 0.15% to 2.6 ± 0.35% upon fermentation, while in the case of soymilk, it was reduced from 9.18 ± 0.04% to 7.43 ± 0.03% after LAB fermentation. During the fermentation of buttermilk or soymilk using LAB strains, exopolysaccharides (EPS) are produced, which act as viscosifying agents. EPS also interact with the milk proteins and form matrices that increase the viscosity of the drinks. These interactions might be responsible for the reduced levels of total solids, carbohydrates, and proteins [21]. Probiotic *Pediococcus acidilactici* species are also capable of synthesizing a variety of EPS as described in the earlier reports [36,37,38].

#### 3.5.3. Variation in Total Phenolic Content

Soymilk has been reported to be the abundant source of phenolics and LAB fermentation has already been reported to be a potent way of enhancing the total phenolic content [21,29]. In the present study, the TPC of the buttermilk and soymilk increased upon fermentation using *P. acidilactici* BD16 (*alaD*^+^). It was observed that upon LAB fermentation, TPC content was enhanced 1.2-fold (from 5.98 ± 0.16 to 6.25 ± 0.04 mg GAE/mL) when compared with the unfermented buttermilk. In the case of soymilk, TPC was approximately 2-fold enhanced from 4.51 ± 0.04 to 7.82 ± 0.05 mg GAE/mL upon LAB fermentation. The literature also supports the findings that the LAB fermentation of soymilk may increase or decrease TPC in comparison to the unfermented drink, depending upon the choice of the LAB strain being used. Fakri, Lim, Musa, Hasan, Adam, and Ramasamy [29] reported that the fermentation of soymilk using *Limosilactobacillus fermentum* LAB 9 strain enhanced the TPC by 10%. In another study, where soybean was fermented using *Lactiplantibacillus plantarum*, TPC increased by 87% in comparison to the unfermented drink [39]. However, few LAB strains such as *Lactobacillus acidophilus*, *Lactobacillus delbrueckii* subsp. *bulgaricus*, and *Lactobacillus casei*, have been reported to reduce the TPC of soymilk with an increased fermentation period [40]. Higher TPC is attributed to the higher free radical scavenging capacity, that eventually enhances the antioxidant potential of the fermented drinks [29].

#### 3.5.4. Variation in Antioxidant Activity

The antioxidant activities (estimated in terms of DPPH radical scavenging ability) of the fermented buttermilk and soy milk enhanced significantly after LAB fermentation. The antioxidant activity of buttermilk increased from 27.2 ± 0.51 to 67.3 ± 0.41%, which is 2.5-fold higher than the unfermented one. In the case of soymilk, the antioxidant activity also showed an increase from 24.3 ± 0.36% to 44.72 ± 0.09% with a 1.8-fold increase in the activity. Soymilk fermentation using LAB strains has also been previously reported to enhance the antioxidant potential in terms of DPPH radical scavenging activity. Marazza and coworkers, in 2012, reported that soymilk fermentation using *Lactobacilli* sp. enhanced the DPPH radical-scavenging activity up to 50% in comparison to the unfermented soymilk. In another study, soymilk fermented for 48 h with a LAB consortium consisting of *Limosilactobacillus fermentum* LAB 10, *Lactiplantibacillus plantarum* LAB 11, and *P. acidilactici* LAB 5 strains exhibited tremendously higher DPPH radical-scavenging activity, approximating 100% value. Fakri and co-workers reported the utilization of *Pediococcus* sp. for the fermentation of soymilk for the first time for enhancing its DPPH radical scavenging activity [29]. According to this study, the enhancement of the antioxidant potential of the LAB fermented drinks could be attributed to the higher isoflavones content, which has been known for protecting the cells from the detrimental effects of free radicals [41]. Tachakittirungrod, Okonogi, and Chowwanapoonpohn [42] also reported that in soymilk fermented using *Lacticaseibacillus rhamnosus* CRL981, aglycones generated during the fermentation process acted as the hydrogen donors, which eventually scavenged DPPH radicals present in the soymilk. Additionally, higher contents of antioxidative compounds such as phenolics and flavonoids have been reported to be responsible for enhancing the free radical scavenging capability of fermented drinks. Therefore, unfermented soybeans or soymilk withlow phenolic content eventually possess low antioxidant potential [30].

#### 3.5.5. Variation in Total Flavonoid Content

LAB fermentation also enhanced the TFC content in the buttermilk and soymilk samples. In the case of fermented buttermilk, TFC values enhanced by 1.7 folds from 3.71 ± 0.29 to 6.19 ± 0.03 mg quercetin equivalents/mL, whereas in the case of fermented soymilk, TFC contents increased by 1.4-fold from 5.76 ± 0.05 to 7.89 ± 0.10 mg quercetin equivalents/mL, which could also be attributed to the enhanced levels of phenolics and antioxidants. Quercetin is a well-known chelator, phytoestrogen, and a free radical scavenger, which could havecontributed to the enhanced DPPH radical scavenging activity of the fermented drinks [43].

### 3.6. Comparison of Sensory Characteristics of the Fermented and Unfermented Buttermilk and Soymilk

Consumer acceptance of soymilk has still been limited due to its undesirable beany aftertaste, caused mainly by the presence of hexanal and pentanal compounds. The formation of these aldehyde compounds has been catalyzed by the lipoxygenase enzyme, which is responsible for the hydroperoxidation of polyunsaturated fatty acids. It has been reported that LAB metabolizes the hexanal and pentanal compounds and converts them into diacetyl and lactic acid during fermentation. Diacetyl is an organic compound produced during LAB fermentation that imparts a buttery, sweet, and soft flavor to dairy food items [21,44].

Other sensory attributes like flavor, odor, mouthfeel, aftertaste, and overall acceptability of the fermented drinks improved considerably after fermentation using the *P. acidilactici* BD16 (*alaD^+^*) strain. In the case of soymilk, homogenized samples were used for hedonic testing and the most important change observed in the sensory attributes of soymilk was a reduction of its beany flavor. Therefore, LAB fermentation of soymilk is being highly recommended to mask its beany flavor and to enhance its commercial value and consumer acceptability.

**Figure 3:**
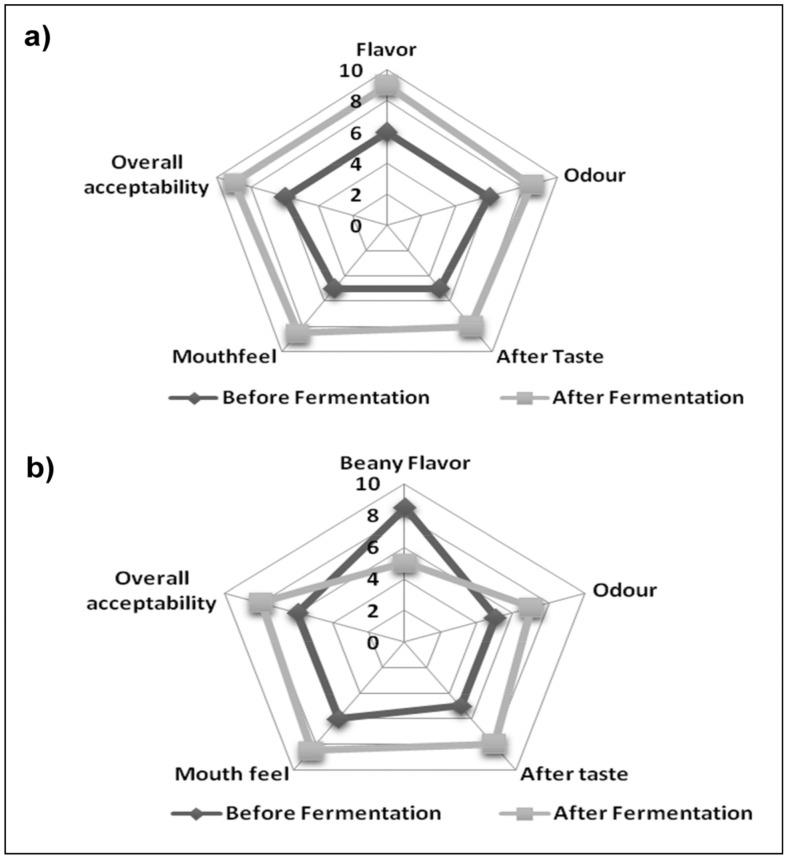
Radar plots showing sensory properties ofthefunctional drinks prepared using *P. acidilactici* BD16 (*alaD^+^*); (**a**) Buttermilk and (**b**) Soymilk. Hedonic scale parameters are graded as 9—Like extremely; 8—Like very much; 7—Like moderately; 6—Like slightly; 5—Neither like nor dislike; 4—Dislike slightly; 3—Dislike moderately; 2—Dislike much; 1—Dislike extremely.

Fermentation using LAB strains has been associated with the development of a creamy mouthfeel, better bulk properties, improved taste and flavor, and higher acceptability among health-conscious consumers. The overall acceptability of the fermented buttermilk was higher as compared to the fermented soymilk due to the formation of soy curd after 8 h of fermentation; however, it can be limited by reducing the duration of fermentation from 6–7 h. Radar graphs clearly depict the improved sensory attributes and better acceptability of the fermented drinks due to the production of LAB-associated metabolites (Figure 3). Thus, the study strengthens the claim of industrial applications of the L-alanine-producing *P. acidilactici* BD16 (*alaD*^+^) strain in enhancing creamy mouthfeel and sweet flavor and masking off-flavors of the drinks, thus opening new vistas for the commercialization of health-promoting LAB-fermented drinks.

### 3.7. GC-MS Based Metabolomic Fingerprinting for the Identification of Significant Bioactive and Heath Health-Promoting Compounds

**Figure 4:**
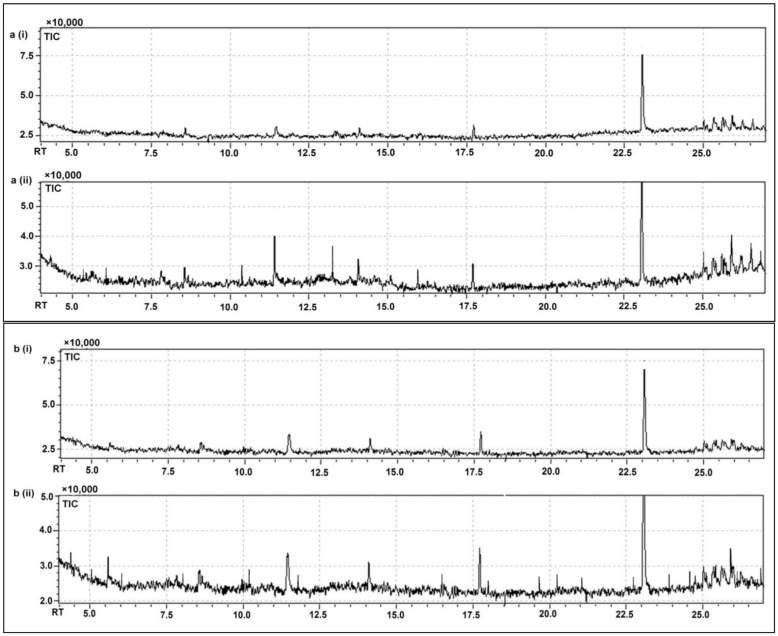
GC-chromatograms of buttermilk; (**a**)/(**i**) before fermentation and (**a**)/(**ii**) after fermentation using *P. acidilactici* BD16 (*alaD*^+^); GC-chromatograms of soymilk; (**b**)/(**i**) before fermentation and (**b**)/(**ii**) after fermentation using *P. acidilactici* BD16 (*alaD*^+^).

GC-MS has been extensively used in metabolomic fingerprinting studies, due to its highly sensitive and specific compound identification ability [45]. The present GC-MS-based study revealed the occurrence of various bioactive compounds, which could account for the improved functional and sensory characteristics of the fermented buttermilk and soymilk using *P. acidilactici* BD16 (*alaD*^+^) (Table 2 and Table 3; Figure 4). LAB fermentation of buttermilk and soymilk eventually increased the number and intensity of many bioactive compounds. The identified compounds belong to diverse classes like alcohols, aldehydes, alkanes, alkenes, amines, amides, carbohydrates, carboxylic acids, ethers, esters, fatty acids, ketones, and organic compounds, which were manually integrated with the previous literature to draw significant inference regarding their role in improving nutritional, functional, and therapeutic attributes of the drinks [3,43,46,47,48,49,50,51,52,53,54,55]. Metabolomic data haverevealed the presence of 32 and 48 compounds in the unfermented and fermented buttermilk, respectively. In the case of soymilk, 37 compounds were detected before and 51 compounds after the LAB fermentation process. The nature, pharmacological significance, and potential applications of the identified compounds were predicted by manual comparison with the available scientific literature. It was found that many compounds detected in the fermented drinks have significant biological activities such as being anti-allergic, anti-Alzheimer’s, anti-arthritic, anti-cancer, anti-convulsant, anti-diabetic, anti-diuretic, anti-depressing, antifungal, anti-gout, anti-inflammatory, antimicrobial, anti-malarial, anti-nephrotoxic, antioxidant, anti-pyretic, anti-protozoal, anti-proliferative, anti-trypanosomal, anti-tubercular, anti-tumor, and anti-viral [3,43,46,47,48,49,50,51,52,53,54,55] as given in detail in Table 2 and Table 3.

Analysis of the untargeted metabolomic fingerprinting data has revealed the crucial role of LAB fermentation in enhancing intensities of certain desirable bioactive and pharmacologically relevant compounds, while reducing the levels of many undesirable and quality detrimental compounds. Fold changes have revealed a considerable decline in the levels of certain undesirable metabolites such as 1-pentadecene, 2-bromopropionic acid, 8-heptadecene, formic acid, and propionic acid upon LAB fermentation, which is responsible for imparting bitterness, rancidity, spoiled flavor, unpleasant odor, and unhealthy attributes to the buttermilk and soymilk. Meanwhile, higher intensities of 1-dodecene, 2-aminononadecane, 4-octen-3-one, acetic acid, benzaldehyde, benzoic acid, chloroacetic acid, heptadecanenitrile, hexadecenoic acid methyl ester, etc., account for the enhancement of aroma, flavor, odor, and taste, the appearance of a creamy texture, and better overall acceptability of the fermented buttermilk and soymilk. Thus, these important metabolic modulations signify the role of the *P. acidilactici* BD16 (*alaD*^+^) strain in improving the quality attributes of the fermented beverages.

In buttermilk, considerably higher levels of 1-dodecene (1.2-fold), 9-octadecene (1.95-fold), benzoic acid (1.5-fold), chloroacetic acid (1.6-fold), colchicine (1.9-fold), Cyclohexadecane (1.8-fold), heptadecanenitrile (1.5-fold), and phosphonic acid (2.2-fold) were observed after fermentation using the *P. acidilactici* BD16 (*alaD*^+^) strain (Table 2). In the case of soymilk, similar metabolic modulations were also observed after LAB fermentation. A considerable increase of 1.2- to 2.05-fold was observed in the case 1-octanamine, 1-tridecene, 2-propenyl decanoate, 3-chloropropionic acid, 3-octadecene, 9-octadecene, decanohydrazide, dichloroacetic acid, lyxitol, octadecatrieonic acid, and phosphonic acid (Table 3). Therefore, the presence of these desirable bioactive compounds highlighted the role of LAB fermentation in improving the quality, functional, sensory, and therapeutic attributes of buttermilk and soymilk.

## 4. Discussion

Soymilk is one of the most popular drinks in Asian and Western countries owing to its extremely nutritive and health-imparting attributes. It contains high-quality proteins, dietary fibers, isoflavones, and unsaturated fatty acids, which are the essential components of a healthy diet. Despite possessing potential health-promoting attributes, its consumption is still being limited due to its undesirable beany flavor and the presence of natural phytic acid, lectins, trypsin inhibitors, and indigestible oligosaccharides [77]. There is a strong need for efficient processing for soymilk preparation that removes these undesirable components and further enhances its nutritive and functional attributes. Heating could be performed to minimize the undesirable flavor caused due to the degradation or oxidation of lipids. It inactivates the lipoxygenase enzyme and thereby reduces the undesirable flavor of soymilk. However, inactivating the lipoxygenase could also lead to the destruction of other nutritional factors such as amino acids, antioxidants, isoflavones, phenolics, and thus, is not recommended in the case of soymilk preparations [2].

Another approach could be the extraction of lipid-based substrates responsible for unwanted flavors; however, complete extraction of lipids is still not feasible. The most effective and reliable approach to improve the quality and flavor of soymilk is by utilizing a LAB fermentation process. The fermentation of soymilk using GRAS-LAB not only enhances its shelf life but also improves its overall acceptability, desirability, nutrition, and health-promoting characteristics [77]. The fermentation of soymilk using galactosidase-producing LAB strains also reduces the raffinose and stachyose contents to improve its digestibility. It has been reported that in the case of soymilk fermentation using *Lactobacillus acidophilus*, the pH of the drink is lowered due to lactic acid accumulation. Moreover, the soymilk fermentation by *L. acidophilus* also masked the beany flavor and enhanced its sensory attributes [5]. Fermentation by bifidobacteria has also been reported to improve the quality of soymilk by enhancing the digestibility of the proteins and by reducing the contents of the unwanted oligosaccharides [78]. In another study, soymilk was fermented for 4 h using *Bifidobacterium animalis* subsp. *lactis* Bb12 to produce multifunctional soymilk having additional benefits of probiotics [77]. In a separate study, functional soymilk was produced by optimizing the soaking (12–24 h) and germination periods (48–96 h) of soybeans. Optimization of the sprouting conditions of soybeans also resulted in increased contents of proteins, amino acids, and total phenolics along with a reduction in the phytic acid content and trypsin inhibitory activity. This has led to the development of high-quality and extremely nutritious soymilk [79]. In addition, fermentation of soymilk containing 3% *w*/*v* okra flour (tofu fermentation residue) using *L. acidophilus* LA3, enhanced the organic acid content as well as its angiotensin-converting enzyme (ACE) inhibitory activity that augmented the health-promoting responses [4]. Soymilk fermentation with probiotic *Limosilactobacillus fermentum* BM-325 strain was also carried out to enhance its ACE inhibitory activity, which increased up to 60%, and a fermentation time of 4 h was proposed to be most suitable to increase the bioactivity of the soymilk [5]. In another study, *Lactobacillus acidophilus*, *Bifidobacterium animalis* subsp. *lactis*, and *Streptococcus salivarius* subsp. *thermophilus* fermented whey was used during the soybean-soaking process for the production of functional soymilk. The soymilk thus produced had higher contents of isoflavones and γ-amino butyric acid (GABA). The pH of the soymilk was reduced from 6.75 to 5.19, whereas the titratable acidity increased from 0.18 to 0.24%. The total solid content of soymilk was reduced substantially from 7.38 to 1.54%,while with the increase in the soaking time of soybeans, isoflavones content decreased significantly but valuable aglycones increased—threefold [26]. Kim, Lee, and Yoo [26] revealed the positive effects of *Lactobacillus delbrueckii* subsp. *bulgaricus* and *Streptococcus salivarius* subsp. *thermophilus* during the fermentation of soymilk by enhancing the amino acids and antioxidants content. However, further post-fermentation sterilization exhibited a detrimental effect on the texture and antioxidant activity probably due to the degradation of certain important but heat-sensitive bioactive compounds that eventually decreased the overall acceptability [21]. Thus, fermenting soymilk with probiotic LAB strains for specified time periods could be highly beneficial in making it a unique multifunctional food.

Similarly, the functional and sensory attributes of buttermilk were also improved after LAB fermentation. Buttermilk is a rich source of vitamins, proteins, and valuable fats. Its balanced nutritional composition also strengthens its nutraceutical and health-promoting claims [9]. Buttermilk has been described in two different types i.e., traditional and cultured, according to their method of preparation. Traditional buttermilk is generally the leftover liquid obtained from churning the LAB-fermented cream into butter [80,81]. However, the cultured buttermilk is obtained by the fermentation of pasteurized skimmed milk using efficient LAB starter cultures such as *Lactococcus lactis* and *Leuconostoc mesenteroides* sp. [82]. In a previous study, the fermentation of buttermilk was carried out using a proteolytic strain of *Lactobacillus helveticus* to prepare a peptide-enriched powder with improved functional and gut-modulating properties [8]. Thus, earlier studies have evidenced that LAB-fermented buttermilk with enhanced nutritional, flavor, and functional properties would be highly impactful in justifying its functional label.

The present study was aimed to impart natural flavor and added nutrition to the soymilk and buttermilk during fermentation using *Pediococcus acidilactici* BD16 (*alaD*^+^). Probiotic *Pediococcus* are generally recognized as safe (GRAS) facultative anaerobes with homo fermentative, Gram-positive, and catalase-negative properties that belong to the bacterial family *Streptococcaceae* and are used extensively in various biotechnological and food applications [83,84]. Currently, the genus *Pediococcus* comprises a group of species including *P. acidilactici*, *P. argentinicus*, *P. cellicola*, *P. damnosus*, *P. ethanolidurans*, *P. inopinatus*, *P. parvulus*, *P. pentosaceus*, *P. siamensis*, and *P. stilesii*. However, only two species i.e., *P. acidilactici* and *P. pentosaceus*, are utilized for the fermentation of dairy products [85]. *P. acidilactici* strains possess interesting properties such as resistance to gastric acids and bile salts. They are also reported to produce many broad-spectrum and heat-resistant antimicrobial peptides known as pediocins, which could control the growth of food spoilage and disease-causing bacteria. Such properties make them attractive candidates for food biopreservation [86]. *P. acidilactici* has already got GRAS status (GRN no. 000171, awarded by Food and Drug Administration); thus, it could be utilized as a probiotic food additive as per WHO guidelines on food and feed additives [20]. It has also got the QPS (The Qualified Presumption of Safety) status recommended by the European Food Safety Authority vide no. EFSA-Q-2018-00268 [87].

Native *P. acidilactici* BD16 MTCC10973 was isolated from a milk product and characterized for its biochemical, molecular, and organoleptic properties for its potential application as a probiotic food and feed additive [20,32,88,89]. Previously, native *P. acidilactici* BD16 was used for vanillin production on a rice bran medium in the presence of ferulic acid as an inducer, with a production yield of 1.269 g/L crude vanillin [88]. To further enhance its vanillin production capacity, a synthetic vanillin gene cassette, bearing *fcs* and *ech* genes encoding feruloyl-CoA synthetase and enoyl-CoA hydratase, was cloned using the shuttle vector pLES003. The pLES003 vector reportedly enhanced the heterologous gene expression and phenolic biotransformation’s in the recombinant strain for improving vanillin production [20]. After metabolic engineering and scale-up of the process, 4 g/L vanillin was recovered from the recombinant cell culture, which is approximately four-fold higher than the yield obtained using native strain.

Later, recombinant *P. acidilactici* BD16 (*fcs*^+^/*ech*^+^) was also explored to enhance flavor and aroma characteristics of the wines during the secondary fermentation process due to increased production of cinnamic acid derivatives. Mass spectral analysis revealed the development of many bioactive phenolic compounds such as apigenin, catechin, coniferyl aldehyde, cyanidin, hydroxybenzoic acids, laricitrin, luteolin, malvidin 3-glucoside, myricetin, naringenin, pelargonin, piceatannol, quercetin, and vanillin during fermentation, which could attribute for the improvement of organoleptic and nutritional properties of the fermented food products. This study also highlighted the role of recombinant *P. acidilactici* BD16 (*fcs*^+^/*ech*^+^) in the production of several phenolic derivatives including vanillin during fermentation, which play a characteristic role in determining the organoleptic properties and thus contributing to the aroma, color, and flavor of the finished product [32].These studies have shown a path for further exploring novel properties of the recombinant *P. acidilactici* BD16 for food-based applications.

Thus, in the present study, the recombinant *P. acidilactici* BD16 (*alaD^+^*) strain (containing the pLES003 plasmid-bearing synthetic *alaD* gene) was utilized as a starter culture candidate for the insitu L-alanine production and for imparting sweetness, nutritional, and health-promoting attributes to the fermented buttermilk and soymilk. Biosafety assessment and evaluation of the probiotic attributes of recombinant *P. acidilactici* BD16 (*alaD*^+^) was also carried out to study its food-based applications. Experimental trials were conducted for evaluation of itshemolytic, gelatinase, and DNase activities, which were not detected in the recombinant strain, thus proving it to be safe for human consumption (unpublished data).

The developed strain greatly influenced the functional, nutritional as well as bioactive potential of the fermented drinks. Enhanced levels of antioxidants, phenolics, flavonoids, and L-alanine after LAB fermentation have been responsible for the improved functional and quality attributes of the developed functional drinks. GC-MS-based metabolomic fingerprinting further persuasively strengthened the argument of the *P. acidilactici* (*alaD^+^*) strain as a starter culture bacterium for the development of functional drinks. However, the authors strongly recommend further research on enhancing the shelf life of fermented functional drinks to boost their market value and consumer acceptability.

## 5. Conclusions

The experimental results convincingly demonstrated the role of *P. acidilactici* BD16 (*alaD*^+^) in improving key functional, nutritional, and sensory attributes of the fermented drinks. GC-MS-based metabolomic fingerprinting further highlighted the presence of significant bioactive compounds in the fermented drinks to support their functional claim. However, the present study still lacks certain experimental validations, which need to be addressed for future industrial applications. Food safety is one of the major concerns when dealing with the development of food products, making food-based applications challenging at many times. Despite GRAS (Generally Recognized as Safe) status, probiotic attributes, and immense industrial potential of *P. acidilactici* BD16, its utilization has still been restricted due to food safety concerns. The draft genome sequence of the strain is available in the GENBANK database, which presents its genetic attributes; however, is still not sufficient to validate its food-based applications. Therefore, complete genetic characterization of the proposed LAB strain is being recommended for identification of the probable virulence characters before its utilization as the starter culture at the industrial level. Moreover, preliminary clinical evaluation needs to be carried out further to report the safety of the fermented drinks for human consumption. In the future, authors will definitely work on overcoming the aforementioned limitations and food safety concerns, to facilitate the application of the proposed LAB strain in the industrial bioprocesses.

## Figures and Tables

**Table 1:** Changes in the physicochemical attributes of the buttermilk and soymilk after LAB fermentation.

S. No.	Physicochemical Attributes	Buttermilk		Soymilk	
		Before Fermentation	After Fermentation	Before Fermentation	After Fermentation
1.	pH	6.8 ± 0.15	3.0 ± 0.25	7.0 ± 0.40	3.5 ± 0.20
2.	Titratable acidity (%)	0.21 ± 0.02	0.87 ± 0.04	0.13 ± 0.02	0.42 ± 0.03
3.	Total solids (%)	2.6 ± 0.1	1.8 ± 0.20	4.9 ± 0.35	2.3 ± 0.25
4.	Moisture content (%)	95.6 ± 0.5	98.3 ± 0.31	93.4 ± 0.26	97.6 ± 0.40
5.	Total carbohydrates (%)	3.5 ± 0.15	2.6 ± 0.35	9.18 ± 0.04	7.43 ± 0.03
6.	Total antioxidants (%)	27.2 ± 0.51	67.3 ± 0.41	24.3 ± 0.36	44.72 ± 0.09
7.	Total flavonoids (mg/mL)	3.71 ± 0.29	6.19 ± 0.03	5.76 ± 0.05	7.89 ± 0.10
8.	Total phenolics (mg/mL)	5.98 ± 0.16	6.25 ± 0.04	4.51 ± 0.04	7.82 ± 0.05
9.	Total protein (mg/mL)	3.06 ± 0.19	2.17 ± 0.05	6.48 ± 0.06	4.21 ± 0.07
10.	L-alanine (g/L)	6.75 ± 0.26	41.5 ± 0.5	8.25 ± 0.18	38.5 ± 0.39

Values are presented as mean ± standard deviation (SD) of triplicates (n = 3), statistical significance was recorded in terms of the *p*-value (significant at a value < 0.005) calculated using the Chisquare test.

**Table 2:** GC-MS-based metabolomic fingerprint data of the functional buttermilk (manually integrated with previously reported biological activities).

S. No.	RT (min)	Compound	Chemical Nature	Occurrence			Biological Activity and Applications	Reference
				BF	AF	FC		
1.	23.06	1,2-Benezenedicarboxylic acid	Dicarboxylic acid	+	+	1.02	Anti-alzheimer; anti-arthritic; anti-cancer; anti-inflammatory; lipoxygenase inhibitor	[54]
2.	7.82	4-Octen-3-One	Ketone	−	+	−	Flavoring agent	[55]
3.	17.71	1-Acetoxynonadecane	Alkane	+	−	−	Anti-microbial	[56]
4.	25.90	1-Bromoeicosane	Alkane	−	+	−	Anti-cancer; anti-inflammatory	[57]
5.	25.90	1-Bromohexadecane	Alkene	−	+	−	Anti-microbial	[58]
6.	8.58	Butafume	Amine	−	+	−	Anti-diabetic; anti-fungal	[55]
7.	17.70	1-Docosene	Alkene	−	+	−	Anti-bacterial; anti-cancer; anti-inflammatory	[49,50]
8.	8.58	1-Dodecene	Alkene	+	+	1.2	Imparts pleasant odor	[55]
9.	8.58	8-Azanonane	Amide	−	+	−	Anti-malarial; anti-protozoal; anti-trypanosomal; used for morphine drug synthesis	[59]
10.	11.45	1H-Indole-2-carboxylic acid	Carboxylic acid	−	+	−	Anti-asthmatic; anti-tussive	[52]
11.	11.40	1-Heptadecene	Alkene	+	−	−	Anti-microbial	[60]
12.	14.07	1-Hexadecanol	Fatty alcohol	−	+	−	Anti-microbial	[49]
13.	11.40	1-Hexadecene	Alkene	−	+	−	Anti-microbial	[58]
14.	8.58	1-Nonadecene	Alkene	+	+	1.04	Anti-cancer; anti-fungal	[60,61]
15.	11.40	1-Pentadecene	Alkene	−	+	−	-	-
16.	25.02	2-Bromopropionic acid	Carboxylic acid	+	+	−	-	-
17.	7.82	2-Hydroxybenzoic acid	Carboxylic acid	−	+	−	Anti-microbial; food preservative; lipase and lipoxygenase inhibitor	[55]
18.	8.58	2-Aminononadecane	Amino alkane	+	+	0.9	Anti-microbial; antioxidant	[55]
19.	25.02	2-Nonyloxirane	-	+	−	−	-	-
20.	25.02	2-Propenyl Decanoate	-	+	+	0.10	Anti-microbial; anti-viral	[47]
21.	25.90	2-Undecenoic acid	Fatty acid	−	+	−	Anti-fungal	[55]
22.	14.10	3-Chloropropionic Acid	Chlorocarboxylic acid	+	−	−	Anti-depressant	[62]
23.	8.58	3-Trifluroacetoxytridecane	Alkane	+	−	−	Anti-nephrotoxic; Antioxidant	[51]
24.	11.40	9-Eicosene	-	−	+	−	anti-hyperglycemic; anti-microbial; antioxidant; cytotoxic; Insecticidal	[49]
25.	11.44	9-Octadecene	Alkene	+	+	1.95	Analgesic; anti-diabetic; anti-inflammatory; anti-microbial; anti-pyretic; antioxidant; anti-tumor	[49]
26.	7.82	Benzaldehyde	Aldehyde	−	+	−	Aromatic and flavoring agent; nitrilase and triacylglycerol lipase inhibitor	[55]
27.	7.82	Benzenecarbothioic acid	Organosulfur compound	−	+	−	-	-
28.	7.82	Benzo-hydrazide	Heterocyclic compound	−	+	−	Anti-cancer; anti-convulsant; anti-microbial; anti-tubercular	[63]
29.	7.82	Benzoic acid	Carboxylic acid	+	+	1.5	Anti-microbial; food preservative; lipase and lipoxygenase inhibitor	[55]
30.	11.44	Cachalot	Fatty alcohol	−	+	−	Anti-microbial	[49]
31.	17.70	Choloroacetic acid	Carboxylic acid	+	+	1.6	Herbicide, food preservative	[55]
32.	25.90	Colchicine	-	+	+	1.9	Anti-gout; anti-inflammatory; inhibits urate crystallization in joints	[55]
33.	14.07	Cyclohexadecane	Alkane	+	+	1.8	Anti-microbial	[64]
34.	11.40	Cyclotetradecane	Cycloalkane	−	+	−	Anti-diuretic; Anti-microbial	[65]
35.	25.91	Cysteamine	Amine/Aminothiol	+	−	−	Anti-nephropathic; radioprotective	[55]
36.	25.90	Decanoic acid	-	+	+	−	-	-
37.	14.07	Dichloroacetic acid	Carboxylic acid, Organochlorine compound	−	+	−	Anti-cancer;pyruvatedehydrogenase kinase inhibitor	[55]
38.	23.06	Diisooctyl phthalate	Carboxylic acid ester	−	+	−	Anti-microbial; anti-fouling	[64]
39.	17.70	Dioctadecyl Phosphonate	-	−	+	−	-	-
40.	25.56	D-Xylitol	Sugar alcohol	+	−	−	Low- calorie sweetener; anti-cancer; anti-diabetic; anti-inflammatory; anti-microbial	[46]
41.	7.825	Ethanone	-	−	+	−	-	-
42.	8.58	Fluoroacetic Acid	Organofluorine compound/haloacetic acid.	+	−	−	Aconitatehydratase inhibitor	[55]
43.	25.24	Glucitol	Sugar alcohol	+	−	−	Low calorie sweetener; diuretic; laxative; cathartic	[55]
44.	17.71	Formic acid	Carboxylic acid	+	+	−0.12	Anti-bacterial;	[66]
45.	25.90	Glucopyranoside	-	+	+	−	-	-
46.	11.40	Heptadecanenitrile	Organic compound	+	+	1.58	Anti-bacterial; anti-cancer; antioxidant	[67]
47.	14.07	Heptadecyl acetate	-	−	+	−	Anti-microbial	[49]
48.	25.02	Hexadecane	Alkane	−	+	−	Anti-bacterial	[64]
49.	25.91	Isochipane B	-	+	−	−	Anti-bacterial	[49]
50.	25.91	Lyxitol	Sugar alcohol	+	−	−	Anti-fungal	[66]
51.	8.58	Heptacosanol	Alcohol	−	+	−	Anti-inflammatory; Anti-thrombotic; cholesterol lowering drug	[68]
52.	23.06	Pyrazinediyl	Heterocyclic compound	−	+	−	Anti-allergic; Anti-cancer; anti-inflammatory; anti-microbial; flavoring agent	[48]
53.	25.90	Pentadecanoic acid methyl ester	+	+	−	−	Anti-microbial	[47]
54.	14.07	Phosphonic acid	Organophosphorus compound	+	+	2.2	Anti-microbial; anti-viral; used in nuclear medicine	[69]
55.	7.825	Propionic acid	Carboxylic acid	+	+	−0.01	Anti-fungal; imparts unpleasant flavor	[55]
56.	25.90	Quercetin	Flavonoid	+	+	1.04	Anti-allergic; anti-bacterial; anti-inflammatory; antioxidant; anti-neoplastic; anti-rheumatic;anti-viral; cytotoxic; aurora kinase, lipoxygenase, cyclooxygenase and protein kinase inhibitor; cardioprotective; cholesterol lowering drug	[43,55]
57.	25.90	Scopolamine- M	-	−	+	−	Anti-cholinergic; anti-inflammatory; gastro-protective; improves CNS functioning	[53]
58.	25.02	Secoyohimban	-	+	−	−	Anti-cancer; antioxidant; anti-microbial	[70]
59.	25.90	Triacontane	Alkane	+	+		Anti-bacterial; Anti-diabetic; Anti-tumor	[64]
60.	17.71	Tricosyl Acetate	-	−	+	−	-	-

Where, + = Present; − = Absent; RT: Retention time; BF: Before fermentation; AF: After fermentation; FC: Fold change.

**Table 3:** GC-MS basedmetabolomic fingerprint data of the functional soymilk (manually integrated with previously reported biological activities).

S. No.	RT (min)	Compound	Chemical Nature	Occurrence			Biological Activity and Applications	Reference
				BF	AF	FC		
1.	23.06	1,2-Benzenedicarboxylic Acid	Dicarboxylic acid	+	+	0.8	Anti-alzheimer; anti-arthritic; anti-cancer; anti-inflammatory; lipoxygenase inhibitor	[54]
2.	25.61	1-Bromododecane	Organobromine compound	+	−	−	Anesthetic	[71]
3.	25.02	1-Bromoeicosane	Alkane	−	+	−	Anti-cancer; Anti-inflammatory	[57]
4.	25.91	1-Decanol	Fatty alcohol	−	+	−	Anti-bacterial; drug delivery agent	[72]
5.	17.71	1-Docosene	Alkene	+	−	−	Anti-bacterial; anti-cancer; anti-inflammatory	[49,50]
6.	11.45	1-Hexadecanol	Fatty alcohol	−	+	−	Anti-microbial	[49]
7.	11.45	1-Hexadecene	Alkene	+	+	−0.12	Anti-microbial	[58]
8.	14.12	1-Nonadecane	Alkane	+	+	−0.10	Anti-microbial	[56]
9.	17.78	1-Octadecanol	Fatty alcohol	−	+	−	Anti-bacterial; antioxidant	[58]
10.	25.91	1-Octanamine	Amine	+	+	1.3	Anti-microbial	[73]
11.	25.02	1-Octanol	Fatty alcohol	+	−	−	Anti-microbial	[72]
12.	14.12	1-Pentadecanol	Fatty alcohol	−	+	−	Anti-microbial	[72]
13.	11.45	1-Pentadecene	Alkene	+	+	−0.5	-	-
14.	14.12	1-Tetradecyl acetate	Ester	−	+	−	-	-
15.	17.71	1-Tricosanol	Alcohol	+	−	−	Anti-microbial	[74]
16.	11.45	1-Tridecene	Alkene	+	+	1.4	Antioxidant	[75]
17.	11.45	2-Aminononadecane	Amino alkane	+	+	0.7	Anti-microbial; antioxidant	[55]
18.	14.12	2-Bromopropionic acid	Carboxylic acid	+	−	−	-	-
19.	25.02	2-Propenyl decanoate	-	+	+	1.2	Anti-microbial; anti-viral	[47]
20.	11.45	3-Chloropropionic acid	Chlorocarboxylic acid	+	+	1.5	Anti-depressant	[62]
21.	17.71	3-Eicosene	-	−	+	−	Anti-microbial; antioxidant; anti-hyperglycemic; cytotoxic; insecticidal	[49]
22.	11.45	3-Octadecene	Alkene	+	+	2.05	Analgesic; anti-diabetic; anti-inflammatory; anti-microbial; antioxidant; anti-pyretic; anti-tumor	[49]
23.	11.45	3-Trifluoracetoxypentadecane	Alkane	+	+	0.78	Antioxidant	[51]
24.	11.45	8-Heptadecene	Alkene	+	−	−	Anti-microbial	[60]
25.	14.10	9,12,15-Octadecadienoic acid	Fatty acid	−	+	−	Anti-acne; anti-androgenic; anti-arthritic; anti-cancer; anti-histaminic; anti-inflammatory; anti-eczemic; cardio-protective; hepatoprotective; 5-alpha reductase inhibitor	[51]
26.	11.45	9-Octadecene	Alkene	+	+	1.56	Anti-bacterial; anti-cancer; antioxidant	[47]
27.	14.12	Acetic acid	Carboxylic acid	+	+	1.10	Anti-microbial; food acidity regulator; aromatic and flavor compound; food preservative	[55]
28.	25.02	Azanonane	Amide	+	+	0.97	Anti-malarial; anti-protozoan; anti-trypanosomal; used for morphine drug synthesis	[59]
29.	23.06	Benzoic acid	Carboxylic acid	+	−	−	Anti-microbial; food preservative; lipase and lipoxygenase inhibitor	[55]
30.	17.71	Cachalot	Fatty alcohol	+	−	−	Anti-microbial	[49]
31.	11.45	Chloroacetic acid	Carboxylic acid	+	+	−0.8	Anti-microbial; herbicide	[55]
32.	14.12	Crodacol	Fatty alcohol	+	+	1.1	Used as ingredient for ophthalmic, rectal and vaginal preparations	[55]
33.	17.74	Cyclohexadecane	Alkane	+	+	0.75	Anti-microbial	[64]
34.	17.71	Cyclotetracosane	-	+	−	−	-	-
35.	14.12	Cyclotetradecane	Cycloalkane	−	+	−	Anti-diuretic; anti-microbial	[65]
36.	25.91	Decanohydrazide	-	+	+	1.3	Anti-bacterial; Anti-convulsant; Anti-inflammatory; Anti-protozoal; tuberculostatic	[76]
37.	25.91	Decanoic acid	-	+	−	−	-	-
38.	14.12	Dichloroacetic acid	Carboxylic acid, Organochlorine compound	+	+	1.4	Anti-cancer; pyruvatedehydrogenase kinase inhibitor	[55]
39.	23.06	Diisooctylphthalate	Carboxylic acid ester	−	+	−	Anti-microbial; anti-fouling	[64]
40.	25.34	D-Xylitol	Sugar alcohol	−	+	−	Low calorie sweetener; anti-cancer; anti-diabetic; anti-inflammatory; anti-microbial	[46]
41.	11.45	Eicosane	-	−	+	−	Anti-microbial; anti-tumor; cytotoxic	[47]
42.	25.61	Eicosenoic acid	Fatty acid	−	+	−	-	-
43.	25.34	Glucitol	Sugar alcohol	−	+	−	Low calorie sweetener; diuretic; laxative; cathartic	[55]
44.	25.02	Glucopyranoside	-	−	+	−	-	-
45.	11.45	Heptadecanenitrile	Organic compound	+	+	0.8	Anti-bacterial; anti-cancer; antioxidant	[67]
46.	17.74	Heptadecylactetate	-	−	+	−	Anti-microbial	[49]
47.	25.91	Hexadecane	Alkane	−	+	−	Anti-bacterial	[64]
48.	11.45	Hexadecenal	Fatty aldehyde	+	+	1.11	Anti-microbial; Anti-viral; promotes apoptosis	[52]
49.	11.45	Hexadecanoic acid, methyl ester	Ester	−	+	−	Antioxidant; hypocholesterolemic; nematicide; flavor compound	[3]
50.	25.61	Hexadecylpyridinium bromide	Pyridinium salt	+	+	0.78	Antiseptic; surfactant; myosin-light-chain kinase inhibitor	[55]
51.	25.91	IdeuteroOctadecanal	Fatty Aldehyde	−	+	−	-	-
52.	25.34	Lyxitol	Sugar alcohol	+	+	1.9	Anti-fungal	[66]
53.	25.02	Octadecatrieonic acid	Fatty acid	+	+	1.2	Anti-cancer; anti-inflammatory; hypocholesterolemic; hepatoprotective	[52]
54.	11.45	Phosphonic acid	Organophosphorus compound	+	+	1.3	Anti-microbial; anti-viral; used in nuclear medicine	[69]
55.	25.61	Propenyl decanoate	-	−	+	−	-	-
56.	25.02	Quercetin	Flavonoid	−	+	−	Anti-allergic; anti-bacterial; anti-inflammatory; antioxidant; anti-neoplastic; anti-rheumatic; anti-viral; cytotoxic; aurora kinase, lipoxygenase, cyclooxygenase and protein kinase inhibitor; cardioprotective; cholesterol lowering drug	[43,55]
57.	25.34	Rhamnitol	Carbohydrate	−	+	−	-	-
58.	25.61	Scopolamine-M	-	−	+	−	Anti-cholinergic; anti-inflammatory; gastro protective; improves CNS functioning	[53]
59.	25.91	Triacontane	Alkane	+	+	1.08	Anti-bacterial; anti-diabetic; anti-tumor	[64]
60.	25.91	Vinyl decanoate	Fatty acid ester	+	−	−	-	-

Where, + = Present; − = Absent; RT: Retention time; BF: Before fermentation; AF: After fermentation; FC: Fold change.

## Data Availability

The data generated from the study is clearly presented and discussed in the manuscript.
